# Equivalent tumor detection for early and late FAPI-46 PET acquisition

**DOI:** 10.1007/s00259-021-05266-7

**Published:** 2021-02-23

**Authors:** J. Ferdinandus, L. Kessler, N. Hirmas, M. Trajkovic-Arsic, R. Hamacher, L. Umutlu, M. Nader, F. Zarrad, M. Weber, W. P. Fendler

**Affiliations:** 1 Department of Nuclear Medicine, University of Duisburg-Essen and German Cancer Consortium (DKTK)-University Hospital Essen, Essen, Germany; 2 Institute for Developmental Cancer Therapeutics, University of Duisburg-Essen and German Cancer Consortium (DKTK)-University Hospital Essen, Essen, Germany; 3 Department of Medical Oncology, West German Cancer Center, University of Duisburg-Essen, Essen, Germany; 4 Department of Diagnostic and Interventional Radiology and Neuroradiology, University Hospital Essen, Essen, Germany

**Keywords:** FAPI, PET, Biodistribution, Fibroblast, Activation, Protein

## Abstract

**Introduction:**

Positron emission tomography (PET) using small ligands of the fibroblast activation protein (FAP) was recently introduced. However, optimal uptake time has not been defined yet. Here, we systematically compare early (~ 10 min p.i.) and late (~ 60 min p.i.) FAPI-46 imaging in patients with various types of cancer.

**Methods:**

This is a retrospective single-institutional study. Imaging was performed at the Essen University Hospital, Germany. A total of 69 patients who underwent dual time-point imaging for either restaging (*n* = 52, 75%) or staging (*n* = 17, 25%) of cancer were included. Patients underwent PET with two acquisitions: early (mean 11 min, SD 4) and late (mean 66 min, SD 9). Mean injected activity was 148 MBq (SD 33).

**Results:**

In total, 400 lesions were detected in 69 patients. Two of 400 (0.5%) lesions were only seen in early time-point imaging but not in late time-point imaging. On a per-patient level, there was no significant difference between SUV_max_ of hottest tumor lesions (Wilcoxon: *P* = 0.73). Organ uptake demonstrated significant early to late decrease in SUVmean (average ∆SUVmean: − 0.48, − 0.14, − 0.27 for gluteus, liver, and mediastinum, respectively; Wilcoxon: *P* < 0.001). On a per-lesion basis, a slight increase of SUV_max_ was observed (average ∆SUV_max_: + 0.4, Wilcoxon: *P* = 0.03).

**Conclusion:**

In conclusion, early (~ 10 min p.i.) versus late (~ 60 min p.i.) FAPI-46 imaging resulted in equivalent lesion uptake and tumor detection. For improved feasibility and scan volume, we implement early FAPI-46 PET in future clinical and research protocols.

**Supplementary Information:**

The online version contains supplementary material available at 10.1007/s00259-021-05266-7.

## Introduction

In adults, fibroblast activation protein (FAP) is expressed at sites of wound healing and fibrosis and in the stroma of many malignancies [1]. High levels of expression on the surface of cancer-surrounding fibroblasts can be observed in many malignancies such as colon [2] and pancreatic carcinoma [3]. In several entities, such as sarcoma, FAP is also expressed on the tumor cell surface [4]. As a result, FAP is a favorable target for cancer imaging.

Recently, a set of small-molecule inhibitors for PET imaging of FAP (FAPI) were introduced [1, 5, 6]. Among these, FAPI-46 exhibited high tumor-to-background uptake ratios enabling oncologic imaging as well as theranostic applications [1]. Dosimetry and biodistribution of [^68^Ga]Ga-FAPI-46 were reported recently in six patients showing high tumor-to-background uptake along with a low equivalent dose of approximately 5.3 mSv for combined 200 MBq [^68^Ga]Ga-FAPI-46 PET with low-dose CT scan. [7]

Although clinical experience with FAP-targeted PET using FAPI-46 (FAPI-46 PET) is growing [8–14], the optimal uptake time has not been defined yet. Here, we systematically compare biodistribution and detection rate between early (~ 10 min p.i.) and late (~ 60 min p.i.) FAPI-46 PET in patients with various types of cancer.

## Materials and methods

### Study design and patients

This is a post hoc analysis of a prospective observational study conducted at the UKE (NCT04571086). A total of 69 study participants who received clinical FAPI-46 PET for cancer imaging until October 2020 were included. All patients underwent dual time-point imaging for either restaging (*n* = 52, 75%) or staging (*n* = 17, 25%) of cancer. Endpoints of the post hoc analyses were (a) lesion detection, (b) biodistribution assessed by SUV in lesions and healthy organs, and (c) uptake in non-tumor pathologies/pitfalls.

The patients gave written informed consent to undergo clinical FAPI-46 PET/CT and were separately consented for study participation. All reported investigations were conducted in accordance with the Declaration of Helsinki and with the national regulations. The study was approved by the local ethics committee (no. 19–8991-BO).

### Image acquisition and interpretation

Scans were performed in craniocaudal direction on a Biograph mMR (Siemens, *n* = 1), Biograph mCT (Siemens, *n* = 20), or Biograph mCT VISION (Siemens, *n* = 48). In each patient, whole-body PET was acquired twice: early (mean 11 min, SD 4) and late (mean 66 min, SD 9). Mean injected activity was 148 MBq (SD 33). All images were interpreted by JF and LK with more than 3 years of experience in PET interpretation. For each dataset, the number of lesions per region (i.e., primary tumor site, nodal, soft tissue metastases, and bone metastases) and per patient was recorded. Spherical volumes of interest (VOI) were employed to determine maximum standardized uptake values (SUV_max_) for up to three tumor lesions per region. For the definition of background, 2-cm-diameter VOIs (4,19 cm^3^) were drawn in mediastinum (bloodpool), liver, and left gluteal muscle, and mean standardized uptake values were (SUVmean) were recorded. Inflammatory uptake and joint uptake, if rated as degenerative rather than malignant, were measured using SUV_max_. The presence of urine tracer retention was recorded for both late and early time-points. Tumor-to-background uptake ratios were calculated as SUV_max_ of the hottest tumor lesion/SUVmean of respective background.

### Statistical analysis

Descriptive statistic methods including calculations of mean, median, and range are used to present continuous data. Frequency and percentage are given for discrete data. Data was tested for Gaussian distribution using Shapiro-Wilk tests. In the case of Gaussian distribution, paired student’s *t* tests were used. In the case of non-Gaussian distribution, nonparametric paired *t* tests (Wilcoxon signed rank tests) were used to compare uptake measurements of tumor lesions, non-tumor lesions, and background SUV values. To compare the frequency of tracer uptake in the ureters, contingency testing using fishers exact test was used. All statistical analysis and graphical presentation of data were performed using R statistics using the ggplot2 package (version 3.4.1, www.r-project.org).

## Results

### Lesion detection

In total, 400 lesions were detected in 69 patients. Patient characteristics are given in Table 1. Lesions were rated as primary tumor site (79/400, 19.8%), lymph node metastasis (86/625, 21.5%), soft tissue metastasis (212/400, 53.0%), or bone metastasis (23/400, 5.8%). Two of 400 (0.5%) of lesions were seen in early time-point imaging but not in late time-point imaging. A patient example is outlined in Fig. 3. These were a hepatic metastasis in one case (see Fig. 3) and a bone metastasis in another case. For both cases, the disease stage did not change, as other lesions of the same regions were visible in both cases. No additional lesions were observed on late imaging. Table 2 summarizes imaging specifications.

**Table 1:** Patient characteristics

*N* = 69	Overall
Age (years)	
Mean (SD)	56.0 (14.1)
Median [min, max]	58.6 [18.7, 79.7]
Sex	
Female	35 (50.7%)
Male	34 (49.3%)
Weight (kg)	
Mean (SD)	76.1 (16.8)
Median [min, max]	76.5 [48.0, 130]
Oncologic diagnosis	
Sarcoma	33 (47.8%)
Pancreatic	18 (26.1%)
NSCLC	7 (10.1%)
Ovarian	4 (5.8%)
Gastrointestinal stromal tumor (GIST)	2 (2.9%)
Thyroid	2 (2.9%)
Cholangiocellular carcinoma	1 (1.4%)
Mesothelioma	1 (1.4%)
Urothelial carcinoma	1 (1.4%)
Purpose of FAPI-46 Scan	
Restaging	52 (75.4%)
Staging	17 (24.6%)

**Table 2:** Imaging specifications

*N* = 69	Overall
Administered activity (MBq)	
Mean (SD)	148 (33.0)
Median [min, max]	146 [58.0, 221]
Early uptake time (minutes)	
Mean (SD)	11.4 (4.16)
Median [min, max]	10.0 [2.00, 34.0]
Late uptake time (minutes)	
Mean (SD)	65.6 (9.03)
Median [min, max]	61.0 [57.0, 92.0]
Anatomical imaging	
Full-dose CT	17 (24.6%)
Low-dose CT	51 (73.9%)
MRI	1 (1.4%)

**Fig. 1:**
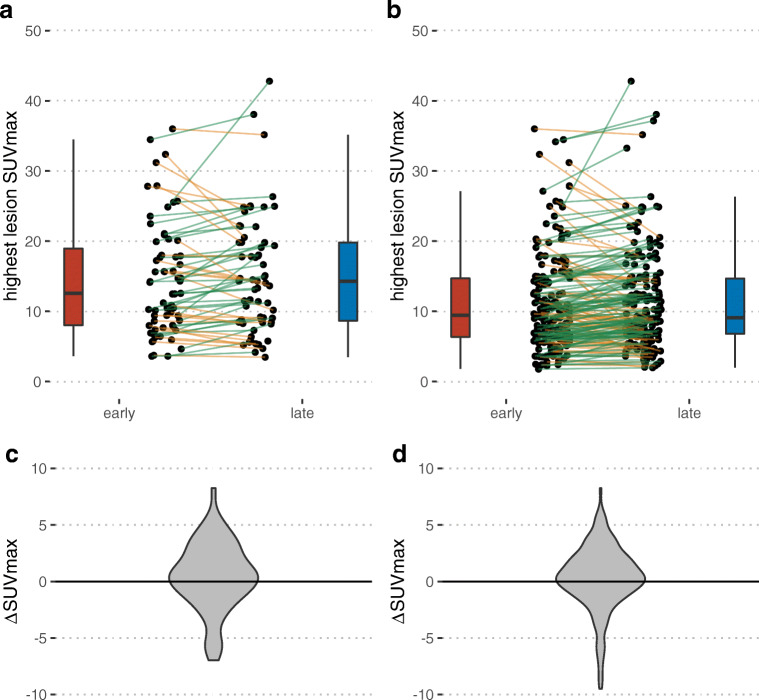
Change of tumor uptake on a per-patient basis (**a** and **c**) and per lesion basis (**b** and **d**). Uptake is shown as individual patient data (dots, lines) and summary box plots. One outlier was removed with change of SUVmax from 61.9 (early) to 89.6 (late)

On a per-patient level, there was no significant difference between SUV_max_ of the hottest tumor lesions (median 14.1 vs 14.3; Wilcoxon: *P* = 0.32; Fig. 1a and b). Stratified by disease site, there was no statistically significant difference between early and late SUV_max_, as demonstrated in Table 3.

**Table 3:** Comparison of FAPI-46 uptake between early and late imaging

*N* = 69	Early	Late	*P*
SUV_max_ primary tumor site			
Mean (SD)	15.2 (10.8)	16.2 (13.8)	
Median [min, max]	12.0 [3.63, 61.9]	14.2 [4.20, 89.6]	0.33
SUV_max_ nodal			
Mean (SD)	11.6 (6.46)	12.2 (6.59)	
Median [min, max]	9.15 [2.96, 23.6]	11.2 [3.11, 25.0]	0.14
SUV_max_ soft tissue metastases			
Mean (SD)	10.9 (7.69)	11.3 (7.90)	
Median [min, max]	9.68 [2.28, 34.2]	9.70 [2.29, 38.0]	0.22
SUV_max_ bone metastases			
Mean (SD)	9.44 (1.96)	8.05 (2.17)	
Median [min, max]	10.1 [6.04, 11.2]	7.89 [5.20, 11.7]	0.16
SUV_max_ inflammatory			
Mean (SD)	7.80 (4.37)	6.22 (3.88)	
Median [min, max]	8.64 [0.00, 17.5]	5.47 [0.00, 15.7]	0.02
SUV_max_ joint			
Mean (SD)	5.75 (2.54)	6.22 (2.39)	
Median [min, max]	4.81 [2.77, 14.7]	5.90 [2.83, 12.0]	0.19
SUV_max_ uterus			
Mean (SD)	11.4 (5.75)	12.4 (6.38)	
Median [min, max]	10.7 [3.62, 28.5]	11.1 [4.60, 31.2]	0.10
SUV_mean_ gluteal background			
Mean (SD)	1.56 (0.449)	1.10 (0.431)	
Median [min, max]	1.46 [0.620, 3.54]	0.980 [0.490, 3.03]	< 0.001
SUV_mean_ liver background			
Mean (SD)	0.866 (0.327)	0.707 (0.298)	
Median [min, max]	0.760 [0.440, 2.11]	0.600 [0.360, 1.51]	< 0.001
SUV_mean_ bloodpool background			
Mean (SD)	1.52 (0.397)	1.22 (0.296)	
Median [min, max]	1.45 [0.910, 3.67]	1.19 [0.790, 2.21]	<0.001
ureter uptake			
No	4 (5.8%)	16 (23.2%)	
Yes	65 (94.2%)	53 (76.8%)	0.004

In total, 172 lesions (three hottest lesions per region) were measured to evaluate the change of SUV_max_ on a per-lesion basis. Here, a slight increase was observed on the later scan (median ∆SUV_max_ = 0.4; Wilcoxon: *P* = 0.03; Fig. 1c and d).

### Background and TBR

**Fig. 2:**
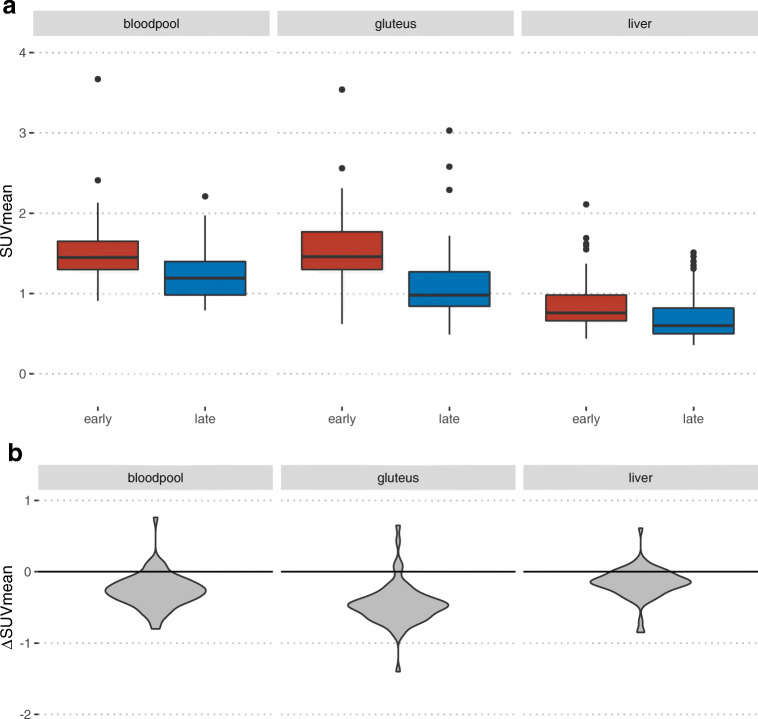
Change of background uptake. **a** A summary of boxplots of SUVmean in bloodpool, gluteus muscle, and liver in early and late acquisition. **b** The distribution of individual differences between SUVmean between early and late imaging

All background measurements showed at late time-point imaging a significant decrease in SUVmean as demonstrated in Fig. 2. Table 4 shows the calculated tumor-to-background ratios (TBR). There was a significant higher tumor/liver, tumor/bloodpool, and tumor/muscle ratio in the later acquisition time (median 15.3 vs 23.3, 9.3 vs 12.3, and 9.4 vs 13.5; Wilcoxon: *P* = 0.001, respectively).

**Table 4:** Tumor-to-background ratios

*N* = 69	Early	Late	*P*
T: liver			
Mean (SD)	19.2 (13.3)	24.5 (15.2)	
Median [min, max]	15.3 [4.01, 72.0]	23.0 [6.25, 87.8]	< 0.001
T: bloodpool			
Mean (SD)	10.5 (6.89)	13.2 (7.75)	
Median [min, max]	9.25 [2.57, 38.0]	12.3 [3.85, 51.8]	< 0.001
T: gluteal muscle			
Mean (SD)	10.5 (7.10)	15.7 (11.8)	
Median [min, max]	9.41 [2.28, 43.0]	13.5 [3.28, 86.1]	< 0.001

**Fig. 3:**
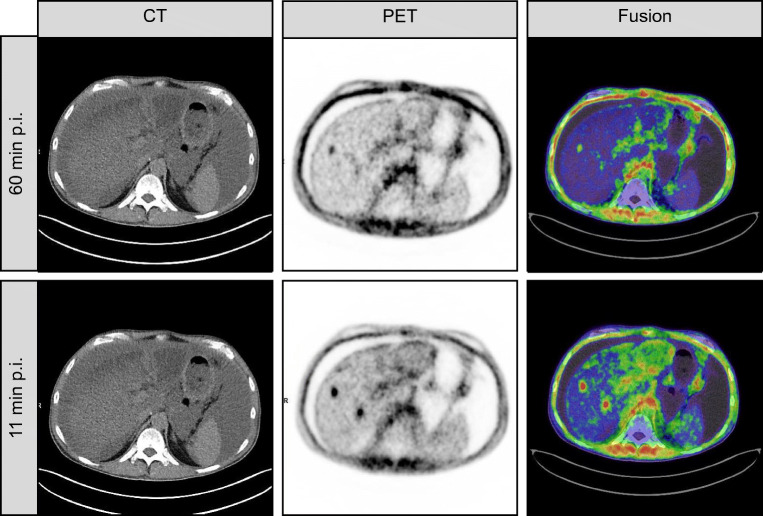
Case example of a patient with discrepant findings between early imaging and late imaging. This is a case example of a 54-year-old patient with metastatic pancreatic cancer. FAPI-46 PET was performed for restaging after resection and de novo peritoneal involvement. FAPI-46 PET performed 11 min after injection revealed two lesions of which one was not noted on late imaging. Increased background uptake is noted due involvement of hepatic viscera with possible intrahepatic cholangitis. Diffuse peritoneal involvement was noted on both scans

Degenerative joint uptake was not significantly different (median 4.8 vs 5.9, student’s *t* test: *P* = 0.19,), whereas inflammatory uptake significantly decreased at the late time-point (median 8.6 vs 5.5, Wilcoxon: *P* = 0.02). An example of the decrease of inflammatory uptake from early to late imaging is outlined in the supplement. Tracer retention in one or both ureters was present in 65/69 (94.2%) vs. 53/69 (76.8%) patients in early vs. late imaging, respectively (Fisher’s test: *P* = 0.004). Table 3 summarizes the results on a per-patient level. Figure 3 illustrates an imaging example of a patient with discrepant findings between early and late imaging.

## Discussion

In this study, we compare detection rate, lesion, and organ uptake for early (~ 10 min p.i.) versus late (~ 60 min p.i.) FAPI-46 PET following intravenous application of [^68^Ga]Ga-labeled FAPI-46. Early versus late PET detection rates were nearly equal. The uptake in organs and non-tumor pathologies was slightly lower for late PET acquisition, however without relevant impact on lesion detection or tumor staging.

Kratochwil et al. previously reported high SUV_max_ values in various tumor entities (mean SUV_max_ of primary 11.5 vs. 10.7 for metastasis) using FAPI-04 [15]. We here observe higher SUV_max_ in tumor lesions (mean SUV_max_ primary 13.4 early vs. 14.3 late), possibly explained by a different cohort and use of FAPI-46 in this analysis. FAPI-46 previously demonstrated higher tumor retention when compared to FAPI-04. [1]

In a dosimetry study on six patients, Meyer et al. observe TBR greater than 10 as early as 10 min p.i. but with increasing values over time [7]. Our data support these observations and confirm the decrease of background signal as well as the moderate increase of tumor uptake on a per-lesion basis and similar magnitude for lesion SUV_max_. As a result, there was a significant increase in TBR for all assessed organs. However, this has not translated into improved lesion detection.

When assessed visually, FAPI-46 PET background uptake is lower than uptake on FDG PET at 10 min p.i. In line with uptake measurements, detection rates were near equal for early versus late acquisition. Interestingly, two discrepant lesions were missed on late time-point imaging but not in early time-point imaging. We thus hypothesize that acquisition between 10 min and 60 min will render near equivalent detection rates, and other time-points in this range might be feasible. Whether there is a role for earlier, possibly dynamic imaging or even very late imaging can be determined in future, explorative studies.

Apart from TBR, uptake in non-tumor pathologies is critical for FAPI-46 PET clinical applications. We observe that uptake labeled as inflammation demonstrated a decrease in intensity from early to late acquisition. This was observed for tumor-associated pancreatitis, which has been reported previously [16]. Thus, late or even two time-point imaging of patients with pancreatic cancer may aid in the discrimination of inflammatory versus malignant uptake. However, in our analysis, none of the pancreatic tumors were missed on early imaging as compared to late imaging. Furthermore, urinary retention was less frequent in late scans as compared to early acquisition. Ureter uptake did not impact lesion detection in our assessment. However, patients with intrapelvic carcinoma or a high likelihood of retroperitoneal lymph node metastasis (i.e., cervical cancer) or equivocal findings in an early scan might also benefit from increased uptake time.

Early image acquisition might improve the feasibility of clinical implementation due to increased scan volumes, simplified workflows, and reduced waiting area space at the nuclear medicine department.

Our study comes with limitations. We have not assessed accuracy as an endpoint and formal lesion validation was not part of this analysis, which may have led to a false classification of lesions. Ultimately prospective trials with lesion validation are needed to determine detection rate, accuracy, impact on management, and theranostic value of FAPI-46 PET.

## Conclusion

In conclusion, intraindividual early versus late FAPI-46 PET acquisition resulted in equivalent detection rates with slight, clinically nonrelevant early to late increase in tumor-to-background uptakes. Due to improved feasibility and scan volume, we implement early FAPI-46 PET in future clinical and research protocols.

## Supplementary information

ESM 1 (DOCX 138 kb)

## References

[1] Loktev A, Lindner T, Burger EM, Altmann A, Giesel F, Kratochwil C (2019). Development of fibroblast activation protein-targeted radiotracers with improved tumor retention. J Nucl Med.

[2] Henry LR, Lee HO, Lee JS, Klein-Szanto A, Watts P, Ross EA (2007). Clinical implications of fibroblast activation protein in patients with colon cancer. Clin Cancer Res.

[3] Cohen SJ, Alpaugh RK, Palazzo I, Meropol NJ, Rogatko A, Xu Z (2008). Fibroblast activation protein and its relationship to clinical outcome in pancreatic adenocarcinoma. Pancreas.

[4] Dohi O, Ohtani H, Hatori M, Sato E, Hosaka M, Nagura H (2009). Histogenesis-specific expression of fibroblast activation protein and dipeptidylpeptidase-IV in human bone and soft tissue tumours. Histopathology.

[5] Loktev A, Lindner T, Mier W, Debus J, Altmann A, Jager D (2018). A tumor-imaging method targeting cancer-associated fibroblasts. J Nucl Med.

[6] Lindner T, Loktev A, Altmann A, Giesel F, Kratochwil C, Debus J (2018). Development of quinoline-based theranostic ligands for the targeting of fibroblast activation protein. J Nucl Med.

[7] Meyer C, Dahlbom M, Lindner T, Vauclin S, Mona C, Slavik R, et al. Radiation dosimetry and biodistribution of (68)Ga-FAPI-46 PET imaging in cancer patients. J Nucl Med. 2019. 10.2967/jnumed.119.236786. 10.2967/jnumed.119.236786 PMC7413240 31836685

[8] Chen H, Pang Y, Wu J, Zhao L, Hao B, Wu J (2020). Comparison of [(68)Ga]Ga-DOTA-FAPI-04 and [(18)F] FDG PET/CT for the diagnosis of primary and metastatic lesions in patients with various types of cancer. Eur J Nucl Med Mol Imaging.

[9] Chen H, Zhao L, Ruan D, Pang Y, Hao B, Dai Y, et al. Usefulness of [(68)Ga]Ga-DOTA-FAPI-04 PET/CT in patients presenting with inconclusive [(18)F]FDG PET/CT findings. Eur J Nucl Med Mol Imaging. 2020. 10.1007/s00259-020-04940-6. 10.1007/s00259-020-04940-6 32588089

[10] Koerber SA, Staudinger F, Kratochwil C, Adeberg S, Haefner MF, Ungerechts G, et al. The role of FAPI-PET/CT for patients with malignancies of the lower gastrointestinal tract - first clinical experience. J Nucl Med. 2020. 10.2967/jnumed.119.237016. 10.2967/jnumed.119.237016 PMC9374030 32060216

[11] Luo Y, Pan Q, Yang H, Peng L, Zhang W, Li F. Fibroblast activation protein targeted PET/CT with (68)Ga-FAPI for imaging IgG4-related disease: comparison to (18)F-FDG PET/CT. J Nucl Med. 2020. 10.2967/jnumed.120.244723. 10.2967/jnumed.120.244723 32513902

[12] Shi X, Xing H, Yang X, Li F, Yao S, Zhang H, et al. Fibroblast imaging of hepatic carcinoma with (68)Ga-FAPI-04 PET/CT: a pilot study in patients with suspected hepatic nodules. Eur J Nucl Med Mol Imaging. 2020. 10.1007/s00259-020-04882-z. 10.1007/s00259-020-04882-z 32468254

[13] Syed M, Flechsig P, Liermann J, Windisch P, Staudinger F, Akbaba S, et al. Fibroblast activation protein inhibitor (FAPI) PET for diagnostics and advanced targeted radiotherapy in head and neck cancers. Eur J Nucl Med Mol Imaging. 2020. 10.1007/s00259-020-04859-y. 10.1007/s00259-020-04859-y PMC7567680 32447444

[14] Zheng J, Yao S (2020). [(68)Ga]Ga-DOTA-FAPI-04 and [(18)F] FDG PET/CT for the diagnosis of primary and metastatic lesions in patients with hepatic cancer. Eur J Nucl Med Mol Imaging.

[15] Kratochwil C, Flechsig P, Lindner T, Abderrahim L, Altmann A, Mier W (2019). (68)Ga-FAPI PET/CT: tracer uptake in 28 different kinds of cancer. J Nucl Med.

[16] Luo Y, Pan Q, Zhang W, Li F (2020). Intense FAPI uptake in inflammation may mask the tumor activity of pancreatic cancer in 68Ga-FAPI PET/CT. Clin Nucl Med.

