# Supplementary material for: Equivalent tumor detection for early and late FAPI-46 PET acquisition
Source: Eur J Nucl Med Mol Imaging. 2021 Feb 23;48(10):3221–7. doi: 10.1007/s00259-021-05266-7 (PMC8426301; doi:10.1007/s00259-021-05266-7)
Supplement: Supplementary file 1 — (DOCX 138 kb) [file 259_2021_5266_MOESM1_ESM.docx]

**Supplementary Figure 1: Case example of a patient with decrease of inflammatory uptake between early imaging and late imaging**


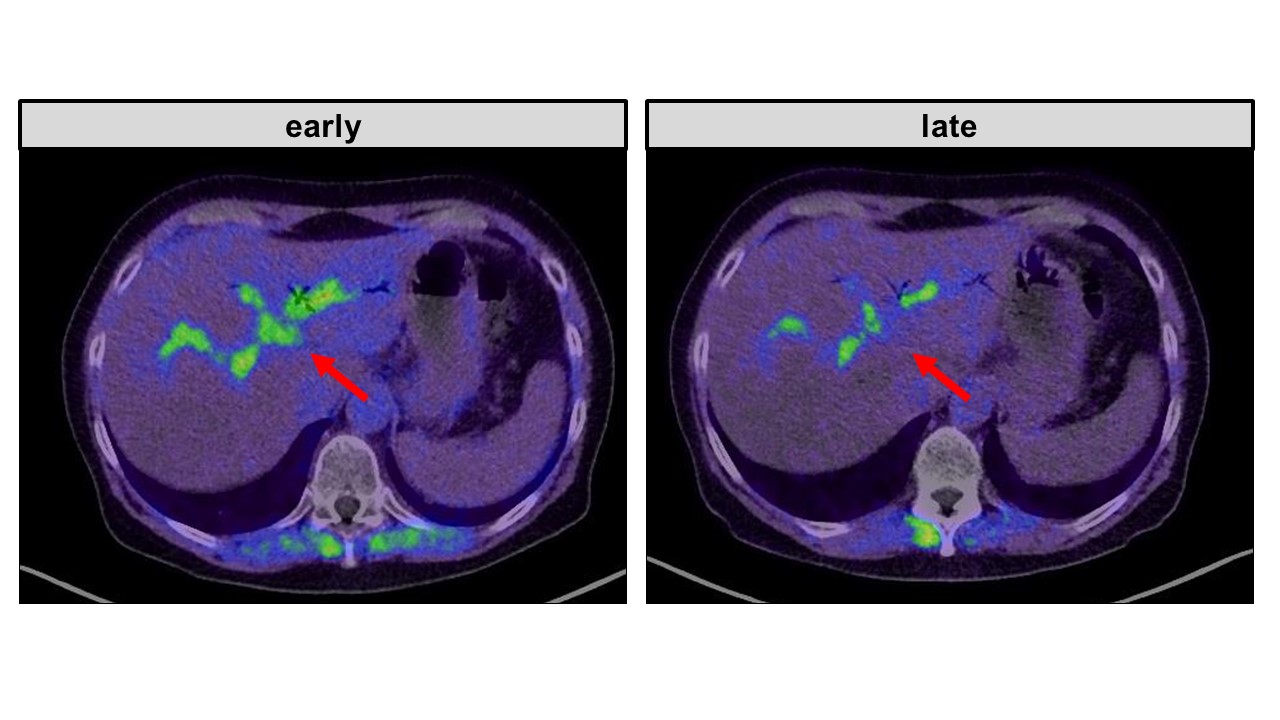


This is a case example of a 62-y/o patient with suspected cholangitis. FAPI-46 PET was performed for re-staging after resection of pancreatic cancer and de-novo peritoneal involvement. FAPI-46 PET revealed aerobilia and surrounding uptake (red arrow), which decreased from early to late imaging (SUVmax 6.2 vs 4.8)
